# Effectiveness of non-specialist delivered psychological interventions on glycemic control and mental health problems in individuals with type 2 diabetes: a systematic review and meta-analysis

**DOI:** 10.1186/s13033-022-00521-2

**Published:** 2022-02-05

**Authors:** Ayodeji D. Oyedeji, Ibrahim Ullah, Scott Weich, Richard Bentall, Andrew Booth

**Affiliations:** 1 School of Health and Related Research, University of Sheffield, Sheffield, S1 4DA UK; 2 Centre for Assistive Technology and Connected Healthcare, School of Health and Related Research, University of Sheffield, Sheffield, S1 4DA UK; 3 Department of Psychology, University of Sheffield, Sheffield, S1 4DA UK

**Keywords:** Type 2 diabetes mellitus, Psychological intervention, Non-specialists, Mental health, Cognitive behavior therapy, Motivational interviewing, Systematic review

## Abstract

**Background:**

Typically, specialist mental health professionals deliver psychological interventions for individuals with poorly controlled type 2 diabetes mellitus (T2DM) and related mental health problems. However, such interventions are not generalizable to low- and middle-income countries, due to the dearth of trained mental health professionals. Individuals with little or no experience in the field of mental health (referred to as non-specialists) may have an important role to play in bridging this treatment gap.

**Aim:**

To synthesise evidence for the effectiveness of non-specialist delivered psychological interventions on glycaemic control and mental health problems in people with T2DM.

**Methods:**

Eight databases and reference lists of previous reviews were systematically searched for randomized controlled trials (RCTs). Outcome measures were glycated hemoglobin (HbA1c), diabetes distress and depression. The Cochrane Collaboration Risk of Bias Tool was used for risk of bias assessment. Data from the included studies were synthesized using narrative synthesis and random effects meta-analysis.

**Results:**

16 RCTs were eligible for inclusion in the systematic review. The 11 studies that were pooled in the meta-analysis demonstrated a reduction in HbA1c in favor of non-specialist delivered psychological interventions when compared with control groups (pooled mean difference = − 0.13; 95% CI − 0.22 to − 0.04, p = 0.005) with high heterogeneity across studies (*I*^2^ = 71%, p = 0.0002). The beneficial effects of the interventions on diabetes distress and depression were not consistent across the different trials.

**Conclusion:**

Non-specialist delivered psychological interventions may be effective in improving HbA1c. These interventions have some promising benefits on diabetes distress and depression, although the findings are inconclusive. More studies of non-specialist delivered psychological interventions are needed in low- and middle-income countries to provide more evidence of the potential effectiveness of these interventions for individuals living with T2DM.

## Background

Type 2 diabetes mellitus (T2DM) is a prevalent and progressive chronic illness, with 463 million people worldwide estimated to be living with this condition and a projected increase to 700 million by 2045 [1]. Most individuals living with T2DM experience different negative emotions and maladaptive behaviours that affects their effort to adjust to the self-management regimen required to maintain optimal glycaemic levels [2]. In recent times, the mental state of individuals living with T2DM has received attention with Lin et al. [2] highlighting the prevalence of depression, diabetes distress (subclinical emotional distress) and anxiety among these individuals. In addition, Fisher et al. [3] and Nefs et al. [4] found that symptoms of depression and diabetes distress persist over time, and for at least 12 months after diabetes diagnosis. Research suggests that there is a bidirectional association in the form of shared biological mechanisms and burden of the condition [5, 6].

Individuals with T2DM experience depression at a rate twice that of the general population [7, 8]. Diabetes distress (defined as the negative feelings, moods and attitudes that individuals living with diabetes experience as they live with, and manage diabetes on a daily basis) a higher prevalence than depression across different settings, ranging from 18 to 64%. [9, 10] In North American and European studies, co-occurring symptoms of diabetes distress and depression in people with diabetes are associated with functional impairment, onset of diabetes-related complications, early mortality, poor adherence to dietary regimen and hyperglycemia [11–14]. Similar findings have been reported in studies in sub-Saharan Africa [15–17], where the co-occurrence of these mental health problems alongside T2DM is associated with reduced quality of life, poor medication adherence, increased healthcare costs and low financial status in individuals.

With increasing numbers of people living with T2DM globally, interventions are needed to simultaneously address mental health problems and improve key diabetes-related outcomes (glycaemic control) in individuals with T2DM. Previous systematic reviews and meta-analyses [18–21], showed that psychological interventions namely cognitive behavior therapy (CBT), client centered therapy (CCT), problem solving therapy (PST), motivational interviewing (MI) and mindfulness, were more effective than usual care in reducing depression and diabetes distress and improving glycaemic control in people with T2DM, with effect sizes ranging from medium to large. Chew et al. [22] and Winkley et al. [23] found a small effect of psychological interventions on HbA1c, with both reviews reporting effect sizes of 0.14 and 0.19 respectively. The small effects on HbA1c in these reviews may be explained by improving standards of usual care for diabetic patients seen in studies conducted in high income settings as well as the good glycaemic control of participants in majority of these studies.

Despite suggestions that psychological interventions can be beneficial in the management of diabetes, with much of the available evidence coming from high income countries and while, there is the likelihood of positive and consistent effects in low- and middle-income countries especially sub-Saharan Africa, this approach cannot be applied in these settings due to shortage of trained mental health professionals. To put that into context, it is estimated that on average, there are 44.8 mental health professionals per 100,000 population in European countries compared with 1.6 per 100,000 population in sub-Saharan African countries [24]. However, there is growing evidence [25–27] that non-specialist such as health professionals (e.g. physicians) and non-health professionals (e.g. university graduates and community health workers) could play important roles in bridging this treatment gap as they have been involved in the detection and treatment of mental health problems. Systematic reviews to evaluate the effects of psychological interventions on glycaemic control and mental health included but did not distinguish between studies which have looked at both specialists and non-specialists delivering the intervention [18, 21, 22]. This makes it difficult to estimate the effectiveness of using non-specialists to deliver psychological interventions for individuals with T2DM. Hence, a review is necessary to synthesise evidence for non-specialist delivered psychologically-informed interventions on the mental health and glycaemic control of individuals with T2DM. Although interventions delivered by non-specialists are likely to be more common in (and relevant to) low- and middle-income settings, studies were not excluded on the basis of setting.

### Objectives

This review was conducted to establish whether psychological interventions delivered by non-specialists (defined as individuals without specialised professional training in the field of mental health) are effective in improving glycaemic control and alleviating mental health problems (depression and diabetes distress), with the secondary aim of identifying which components of these interventions were likely to be important in achieving these outcomes.

## Methods

### Protocol and registration

The protocol of this review is available from PROSPERO database (Registration ID: CRD42020176738). This review was reported in line with the Preferred Reporting Items for Systematic Reviews and Meta-Analyses (PRISMA) guidelines [28].

### Eligibility criteria

Eligibility criteria included randomised controlled trials (RCTs) of non-specialist delivered psychological interventions for individuals (18 years and above) with a clinical diagnosis of type 2 diabetes.

Interventions were classified as psychological if they met the following criteria: (i) at least one part of the intervention was guided by established psychological principles and techniques; (ii) it involved interpersonal interaction between therapist and patient, such that the patient plays an active role in the intervention; (iii) intervention was aimed, either exclusively or in part at improving mental health outcomes. Examples of established psychological therapies are cognitive behavioural therapy (CBT), behaviour therapy, mindfulness and problem-solving therapy (PST). For interventions not explicitly described as psychological, authors were contacted for further information. Studies involving combined or collaborative methods of treatment were included (eg CBT or behaviour therapy combined with diabetes education).

‘Non-specialist’ providers were defined as individuals who have not received intensive professional specialist training in the field of mental health. These included health and social care professionals (doctors, nurses and other allied health professionals). This category also included individuals who have undergone some training in the field of mental health such as undergraduate modules or brief introductory courses in mental health. Non-health professionals such as community health workers, peers, students were considered for inclusion as non-specialist providers as they are involved at the community level and have a significant role to play especially in the detection and treatment of mental health problems as well as improving access to mental health care [29, 30]. Non-specialist providers do not include mental health professionals such as psychiatrists, psychologists, psychiatric nurses and social workers. Comparators (control conditions) were usual care, waitlist and diabetes education. Co-primary outcomes were glycaemic control (change in HbA1c) and depression and/or diabetes distress as measured using validated tools. With regards to depression, diagnostic and symptom severity tools were considered appropriate for inclusion. Studies in which diabetes distress was the mental health outcome were included where this was measured by either Problem Areas in Diabetes scale (PAID-5 or PAID-20) or Diabetes Distress Scale (DDS-17). Non-English articles were omitted based on the linguistic ability of the author.

### Information sources

The following databases were searched in retrieving studies: Ovid PsycINFO, Ovid MEDLINE, Ovid EMBASE, Ovid CINAHL, Cochrane Central Register of Controlled Trials. Websites such as www.clincialtrials.gov, www.globalhealthlibrary.net, and www.who.int/trialsearch were searched for trials that have been completed and their results, as well as reference lists of similar reviews.

### Search strategy

The ‘non-specialist’ search strategies from a review of mental health treatments delivered by non-specialist health workers [31] were used in this review. In addition to this, a combination of keywords, wildcards and relevant truncation related to type 2 diabetes mellitus, depression and diabetes distress was used. Ongoing trials were excluded from this review. There was no limitation on year of publication. A preliminary search was conducted on 19 September 2019 and the final search was conducted on 5 August 2020. The search strategies are shown in Appendix 1.

### Study selection

Reference management software (Mendeley) was used to compile results from the databases and exclude duplicate references. After screening titles and abstracts of retrieved studies, full text of potentially eligible studies was examined for inclusion, with exclusion reasons recorded. Titles and abstracts of retrieved studies were initially screened against the eligibility criteria by the primary author (AO). The second author (IU) independently reviewed a random 10% percent of title, abstracts and full text studies to ensure that there is no incorrect exclusion of relevant studies [32]. In the selection process, consensus was reached through discussion. In the event that consensus was not reached, one of the additional reviewers (SW,RB) was called upon to make the final decision.

### Data extraction

The lead author (AO) used a standardized data collection form (based on Cochrane collaboration data collection form for RCTs) to extract necessary information from included studies, piloted tested it on five randomly-selected included studies with the second author (IU) and refined it accordingly. Data was extracted from each included trial on study design, country, mean age of participants, sample size, duration of T2DM, cadre/choice/title of non-specialist, intervention characteristics and outcomes. Authors of included studies were contacted for missing data.

### Risk of bias and quality assessment

The Cochrane Collaboration Risk of Bias Tool was used to ascertain risk of bias in included studies [33]. The lead author assessed the included studies using the risk of bias tool and a random 10% of the included studies was extracted and assessed independently by the second author (IU). Consensus was reached through discussion. In the event that consensus was not reached, one of the additional reviewers (SW, RB) was called upon to make the final decision. The Grading of Recommendations, Assessment, Development and Evaluations (GRADE) approach was used to assess the quality of evidence for each outcome, which takes into account issues related to internal validity (risk of bias, inconsistency, imprecision, publication bias) and external validity (directness of results). Certainty in the evidence from included studies was rated down from ‘high quality’ by one level for serious (or by two for very serious) study limitations as specified in the GRADE domains (Appendix 3).

### Data synthesis

Data from included studies were pooled in meta-analysis and synthesized using the Review Manager (v5.3). Where statistical pooling was not possible, findings were analysed narratively. Significant diversity was expected in the included studies and as such, random-effects meta-analysis was performed, and assessment of heterogeneity was by chi-squared and Higgins’ *I*^2^ test. In the event that there were adequate number of studies, subgroup analyses by category of non-specialist providers (health professionals and non-health professionals) and intervention characteristics were carried out to check if the intervention effect varied. Risk of publication bias was determined based on visual inspection of a funnel plot. Effect sizes were calculated as standardized mean difference (SMD) for continuous outcome variables and were classified as small effect (0.2), medium effect (0.4) and large effect (0.8) [34].

## Results

**Fig. 1:**
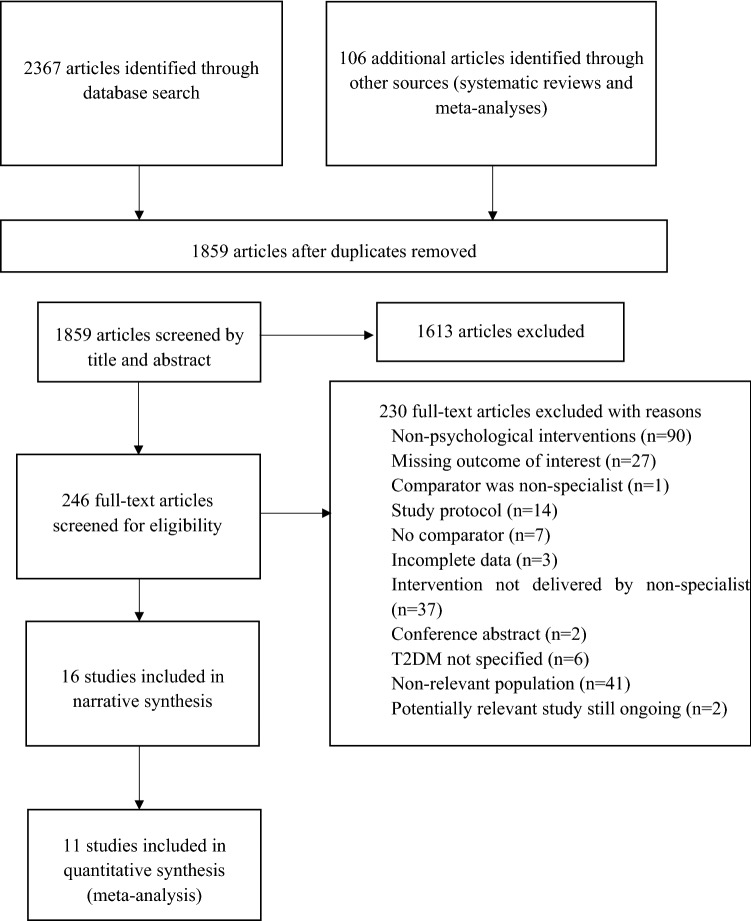
PRISMA diagram showing process of study selection

Initial electronic searches generated 2367 results before elimination of duplicates, with 106 additional references identified through reference lists of systematic reviews and meta-analyses. 1613 studies were excluded after title and abstract screening. There was 81% agreement in identifying abstracts for full retrieval (Cohen’s kappa = 0.81). Disagreements were discussed and when it was not possible to meet consensus, one of the additional reviewers was consulted. 248 full text studies were reviewed., with 16 studies included in the final review (Fig. 1).

### Study characteristics

The 16 included studies were all conducted in high income countries: Germany (n = 1), Netherlands (n = 3), Taiwan (n = 1), UK (n = 2) and USA (n = 9). The characteristics of the studies are summarised in Table 1. Two authors responded to requests for additional information. In total, 4863 participants were involved in the studies in this review and sample sizes ranged from 53 to 1299 participants. A total of eleven RCTs (n = 1940) were included in the meta-analysis and sample sizes ranged from 53 to 545 participants. Twelve studies [35, 37, 39–44, 46, 47, 49, 50] had a two-arm design; two [36, 38] had a three-arm design; two [45, 48] had a four-arm design. Mean age of participants was between 50 to 70.7 years and mean duration of diabetes was between 2.7 ± 3.0 and 10.5 ± 8.3 years. One study [36] did not report mean age of participants. Four trials [36, 39, 41, 47] did not report mean duration of diabetes. There were more female than male participants in 8 studies, [38, 39, 41, 43, 44, 46–48] with one study [50] focusing on only females.

**Table 1:** Characteristics of included studies

Study, design and country	Mean age of participants in years (SD), % of males and females	Sample size, *N* of conditions, control group	Duration of diabetes [mean years (SD)]	Cadre of non-specialist (mode of delivery)	Intervention description (follow-up)	Outcomes measures of relevance [mean baseline scores (SD)]	Results
Chiu et al. [35] RCT, Taiwan	64.6 (8.9) 51.7% males and 48.3% females	N = 174. Two conditions, Usual care	10.5 (8.3)	Research assistants (Telephone)	Focused on T2DM individuals aged 50 years and above with occasional distress or minor depressive symptoms. Individuals received 3–4 CBT sessions consisting of reattribution technique over the phone lasting 30–60 min for 6 weeks. (Follow up at 1-month for all outcome measures plus 3- and 8-month follow up for HbA1c)	*Diabetes distress* Intervention: 5.6 (7.6) Control: 5.4 *Depression* Intervention: 3.2 Control: 3.7 (4.8) *HbA1c (%)* Intervention: 7.6 Control: 7.7 (1.3)	*Diabetes distress:* No significant difference between intervention and control group at the end of intervention and 1 month postintervention. *Depression:* No significant difference between intervention and control group at the end of intervention and 1 month postintervention***. ****HbA1c (%)*: No significant difference in reduction of HbA1c levels between intervention and control group at the end of the intervention and at follow-up periods. Participants in intervention with HbA1c < 8% significantly reduced HbA1c levels compared to control group 3 months post intervention. Reduction sustained at 8-months follow-up
Dale et al. [36] RCT, UK	55.4% males and 44.6% females	N = 231. Three conditions, Usual care	–	Peers and Diabetes nurses (Telephone)	Targeted at individuals with raised HbA1c levels. It consisted of 6 individual motivational interviewing sessions for 6 months. (No follow-up)	*Diabetes distress* Intervention 1: 14.6 (12.7) Intervention 2: 22.7 (18.8) Control: 19.8 (15.5) *HbA1c* Intervention 1: 8.4 (1.1) Intervention 2: 8.9 (1.5) Control: 8.7 (1.3)	*Diabetes distress:* No statistically significant difference between the three groups. *HbA1c (%):* No statistically siginificant difference in HbA1c between the three groups
Dobler et al. [37] RCT, Germany	52 (5.5) 70% males and 30% females	N = 199. Two conditions, Usual care	Intervention: 8.7 (6.6) Control: 9.6 (5.9)	Non-medical dietitians (Telephone)	Targeted at individuals who are German speakers. It consisted of 12 individual motivational interview sessions and problem-solving therapy for 12 months. (No follow-up)	*Diabetes distress* Intervention: 33.2 (19.5) Control: 38.0 (17.5) *Depression* Intervention: 9.2 (5.2) Control: 10.1 (6.3) *HbA1c (%***)** Intervention: 7.8 (1.7) Control: 7.6 (1.4)	*Diabetes distress:* Decreases in PAID scores for diabetes distress post intervention in intervention group by 4.77% and control group by 1.4% were not significant. *Depression*: Significant decreases in PHQ scores in intervention group compared to control group postintervention. *HbA1c (%):* HbA1c scores decreased significantly by 0.68 in intervention group and increased by 0.12 in control group
Fisher et al. [38] RCT, USA	56.11 (9.55) 46.2% males and 53.8% females	N = 392. Three conditions, Diabetes education	6.90 (5.93)	Non-professional college graduates (Web-based, Telephone, In person)	Aimed at distressed, non-clinically depressed T2DM individuals and lasted for 12 months. CASM was a web-based self management intervention for 40 min. Participants in CASM received telephone calls at 8 different time intervals for 15 min. CAPS was an in-person PST combined with CASM for 60 min. Participants in CAPS received a review of PST steps (booster session) at month 5. (Follow-up at 4-months and 12-months)	*Diabetes distres***s** CASM: 2.37 (0.86) CAPS: 2.38 (0.89) LA: 2.48 (0.95) *HbA1c (%)* CASM: 7.45 (1.5) CAPS: 7.34 (1.6) LA: 7.45 (1.7)	*Diabetes distress***:** Significant decrease observed in the 3 groups (CASM, CAPS and LA) and was maintained at follow-up, as participants in CAPS reported significantly greater reduction in diabetes distress. *HbA1c (%)*: For the 3 groups (CASM, CAPS and LA), there was no significant reduction at posttreatment and at follow-up
Gabbay et al. [39] RCT, USA	Intervention: 58 (11.41) Control: 58 (11.34) 42% males and 58% females	N = 545. Two conditions, Usual care	–	Nurses (In person, Telephone, E-mail)	Targeted at T2DM individuals with high risk of developing cardiovascular complications. It consisted of 2–9 Individual motivational interview sessions for 1 h for 24 months. Telephone and e-mail was used in between face to face visits. (No follow-up)	*Diabetes distress* Intervention: 29 (22.64) Control: 29 (24.32) *Depression* Intervention: 14 (14.76) Control: 15 (15.24) *HbA1c (%)* Intervention: 8.82 (2.38) Control: 9.05 (2.27)	*Diabetes distress:* No significant difference between participants in the intervention group and control group at the end of the intervention. *Depression:* Significant reduction in CES-D scores for depression in participants in the intervention group compared to the control group at the end of the intervention. *HbA1c (%)***:** No significant difference in HbA1c decline between both groups as HbA1c reduced in intervention group and in control group
Heinrich et al. [40] RCT, Netherlands	59 (5.27) 55.1% males and 44.9% females	N = 584. Two conditions, Usual care	26.4% had diabetes for less than 1 year 47% had diabetes between 2 and 3 years 26.4% had diabetes between 4 and 5 years	Nurses (In person, web-based)	Aimed at T2DM individuals between 40 and 70 years with diabetes duration of less than 5 years. Individuals received 3 face to face MI sessions for 20 min in conjuction with a web-based educational programme within 12 months. (Follow-up at 1-year and 2 years)	*Diabetes distress* Intervention: 16.83 (13.32) Control: 16.98 (13.92) *HbA1c (%)* Intervention: 6.49 (0.85) Control: 6.51 (0.74)	*Diabetes distress:* There was no significant difference between the intervention and control group at the end of the intervention and at 1 and 2-year follow up. *HbA1c (%):* There was no significant difference between the intervention and control group
Inouye et al. [41] RCT, USA	57.3 (10.9) 45.4% males and 54.6% females	N = 207. Two conditions, Diabetes education	–	Research assistants (In Person)	Aimed at T2DM individuals. The group CBT sessions consisted of biofeedback assisted relaxation, cognitive restructuring, problem solving, contracting, behavioral rehearsal and reinforcements. The group met for six successive weeks for 1–2 h and group size ranged between 2 to 6 individuals. (Follow-up at 12 months)	*Depression*^b^ Intervention: 10.83 ± 0.83 Control: 9.68 ± 0.83 *HbA1c (%)*^*b*^ Intervention: 8.1 ± 0.2 Control: 7.8 ± 0.2	*Depression:* Intervention group had improved in CES-D depressive symptom scores compared to the control group at the end of the study. The improvements were not sustained at follow-up *HbA1c (%):* Differences observed in the intervention group and control group at post-intervention were not statistically significant. However, the scores increased at follow up for intervention and control group
Kim et al. [42] RCT, USA	56.9% males and 43.1% females	N = 250. Two conditions, Waitlist	8.5	Nurses and Community health workers (In Person, Telephone)	Targeted at Korean Americans with poor glycemic control. It consisted of group and individual sessions. The group sessions involved 2-h sessions of CBT techniques (problem solving and cognitive reframing) and diabetes education over the course of 6 weeks. The individual sessions involved 11 sessions of motivational interviewing ranging from 15–45 min. (No follow-up)	*Depression*^b^ Intervention: 5.3 (0.5) Control: 5.4 (0.5) *HbA1c*^b^ Intervention: 8.9 (0.2) Control: 8.8 (0.2)	*Depression:* Control group experienced significant improvement in depressive symptom scores compared to the intervention group at the end of the study. *HbA1c (%)***:** Statistically signifiacnt difference in HbA1c levels between both groups, with clinically significant reductions observed in the intervention group than in the control group
Lamers et al. [43] RCT, Netherlands	Intervention: 70.7 (6.6) Control: 69.7 (6.6) 49% males and 51% females	N = 208. Two conditions, Usual care	Intervention: 8.2 (8.8) Control: 9.8 (9.1)	Nurses (In Person)	It was aimed at T2DM individuals aged 60 years and above with minor to moderate depression and consisted of individual cognitive behaviour therapy elements combined with self-management techniques for 3 months. Participants received 2–10 visits lasting 60 min–90 min over a period of 3 months. (Follow-up at 1-week, 3-months and 9-months)	*Diabetes distress* Intervention: 22.6 (20.5) Control: 23.4 (19.5) *HbA1c (%)* Intervention: 7.5 (1.2) Control: 7.2 (1.4)	*Diabetes distress:* Non-significant difference observed in PAID scores for both groups postintervention at 1 week, 3 months and 9 months. *HbA1c (%):* Non-significant differences observed between both groups postintervention at 1 week and 3 months. Significant improvement between the two groups at 9 months as participants in the intervention group had reduced HbA1c level (7.3%) when compared with increased HbA1c level in the control group (7.8%)
Sacco et al. [44] RCT, USA	52 (8.6) 42% males and 58% females	N = 62. Two conditions, Usual care	9.5 (7.2)	Undergraduate students (Telephone)	Focused on T2DM individuals with poor glycemic control. The individual CBT sessions consisted of activity rescheduling and behavioral experiments for 6 months. Participants received one phone call per week for the first 3 months and one biweekly call for the remaining 3 months. Phone calls lasted 15–20 min. (No follow-up)	*Depression* Intervention: 16.32 (6.60) Control: 16.45 (6.77) *HbA1c (%)* Intervention: 8.4 (1.37) Control: 8.5 (2.01)	*Depression:* Significant reduction in PHQ depressive symptom scores in participants in the intervention group compared with those in the control. *HbA1c (%):* No significant difference between intervention and control group as both groups reported reduced HbA1c levels
Simmons et al. [45] cRCT, UK	Group support: 65.2 (10.2) Individual support: 65.2 (8.9) Combined group and individual support: 65.3 (9.3) Control: 64.6 (10.3), 59.3% 60.4% males and 39.6% females	N = 1299. Four conditions, Usual care	Group support: 7.0 (3.0–12.0)^a^ Individual support: 7.0 (3.0–12.0)^a^ Combined group and individual support: 6.0 (3.0–11.0)^a^ Control group: 6.5 (3.0–12.0)^a^	Peers (In person, Telephone)	Focused on participants with T2DM for at least 12 months. It consisted of motivational interviewing techniques for 8- 12 months received in group, individual or combined group and individual sessions. The duration of the individual and group sessions were 60 min and 90 min respectively. (No follow-up)	*Diabetes distress* Group support: 6.27 (4.22) Individual support: 6.53 (4.12) Combined group and individual support: 6.71 (4.27) Control: 6.61 (4.05) *Depression* Group support: 4.49 (4.92) Individual support: 4.39 (5.13) Combined group and individual support: 4.59 (4.60) Control: 4.49 (5.01) *HbA1c (%)* Group support: 7.5 (1.3) Individual support: 7.4 (1.3) Combined group and individual support: 7.3 (1.3) Control: 7.3 (1.3)	*Diabetes distress*: Participants in the individual support group had a significant reduction in diabetes distress compared to participants in the group interventions *Depression:* No significant difference between participants in the individual support intervention and group interventions with respect to reducing depressive symptoms. *HbA1c (%)*: No significant diffeence between participants in the individual support intervention and group support intervention. In participants with HbA1c above 8%, no significant difference between the individual and group support intervention with respect to reducing HbA1c levels
Spencer et al. [46] RCT, USA	Intervention: 50 (47, 52)^c^ Delayed group: 55 (53, 57)^c^ 29% males and 71% females	N = 164. Two conditions, Waitlist	Intervention: 8 (6, 9)^c^ Delayed group: 9 (7, 11)^c^	Community Health Workers (In person, Telephone)	Aimed at African American and Latino individuals with T2DM. It consisted of combination of diabetes education classes and motivational interviewing sessions. 11 group sessions of motivational interviewing and diabetes education lasting for 2 h were delivered every 2 weeks. The duration of the intervention was 6 months. (Follow-up at 6-months for intervention group)	*Diabetes distress* Intervention: 23.8 (18.7, 29.0)^c^ Delayed group: 25.9 (21.2, 30.6)^c^ *Depression* Intervention: 5.2 (3.9. 6.5)^c^ Delayed group: 5.0 (4.0, 5.9)^c^ *HbA1c (%)* Intervention: 8.6 (8.0, 9.1)^c^ Delayed group: 8.5 (8.0, 8.9)^c^	*Diabetes distress:* Significant greater reduction in PAID scores in participants in the intervention group compared to the control control at end of the intervention. *Depression*: No significant changes in PHQ depressive symptom scores between both groups. *HbA1c (%)*: Participants in the intervention group improved significantly in mean change in HbA1c values compared with the delayed group
Wagner et al. [47] RCT, USA	Intervention: 60.0 (11.2) Control: 60.8 (12.1) 27.1% males and 72.9% females	N = 107. Two conditions, Diabetes education	–	Community Health Worker (In person)	Targeted at individuals with T2DM diagnosis of 6 months or more than and glycemic level above 7.0% in the past one year. It consisted of combination of techniques of CBT and mindfulness therapy in addition to diabetes education. The intervention comprised of 8 groups sessions for 2 h over 8–10 weeks. Participants in the group ranged from 9 to 16. (Follow-up at 3-months)	*Diabetes distress* Intervention: 7.9 (6.7) Control: 8.1 (6.3) *Depression* Intervention: 6.7 (5.9) Control: 5.3 (4.4) *HbA1c (%)* Intervention: 8.5 (1.4) Control: 8.6 (1.9)	*Diabetes distress*: No significant difference between intervention and control group with diabetes distress decreasing in both groups. *Depression*: Significant reduction in depressive symptoms in participants in the intervention group compared with the increase in depressive symptoms observed in the control group. *HbA1c (%)*: No significant difference between intervention and control group postintervention and 3 months follow-up
Welch et al. [48] RCT, USA	55.7 (10.2) 59.1% and 40.9%	N = 234. Four conditions, Diabetes education	8.2 (7.0)	Diabetes educators	Aimed at diabetic individuals between the ages of 30 to 70 years with poorly controlled diabetes above 7.5%. Individuals received four sessions of MI plus diabetes education for 6 months. The first session was one hour and the remaining sessions were for 30 min. (No follow-up)	*Diabetes distress* Intervention 1: 40.5 (23.3) Intervention 2: 41.9 (22.4) Control 1: 43.4 (25.0) Control 2: 42.5 (23.6) *Depression* Intervention 1: 19.1 (9.0) Intervention 2: 18.9 (8.7) Control 1: 19.9 (9.3) Control 2: 18.6 (10.9) *HbA1c (%)* Intervention 1: 9.1 (1.5) Intervention 2: 8.8 (1.0) Control 1: 8.8 (1.3) Control 2: 8.9 (1.62)	*Diabetes distress*: No significant difference between intervention and control groups *Depression*: No significant difference between intervention and control groups. *HbA1c (%)*: Participants in intervention had a mean change in HbA1c that was significantly lower than control group
Welschen et al. [49] RCT, Netherlands	Intervention: 60.5 (9.4) Control: 61.2 (8.8) 61.7% males and 38.3% females	N = 154. Two conditions, Usual care	Intervention: 7.6 (5.0) Control: 7.8 (6.1)	Dietitians and Diabetes nurses (In person)	Targeted at T2DM individuals with glycemic level higher than 7%. It consisted of 3–6 individual CBT sessions of 30 min. (Follow-up at 6-months and 12-months)	*Depression* Intervention: 11.1 (8.1) Control: 9.6 (8.2) *HbA1c (%)* Intervention: 6.8(1.0) Control: 6.7 (1.0)	*Depression*: Participants in the intervention group reported significant decrease in depressive symptoms compared with the control group. This was not sustained 6 months later. *HbA1c (%)*: No significant difference between intervention and control group at 6 months follow-up and 12 months follow-up
Whittemore et al. [50] RCT, USA	57.6 (10.9) Participants were females	N = 53. Two conditions, Usual care	2.7 (3.0)	Nurse (In person, telephone)	Aimed at T2DM women with glycemic level higher than 7%. It consisted of 6 individual sessions of MI in addition to diabetes education for 1 h lasting for 6 months. Two telephone calls were provided between session 5 and 6. (No follow-up)	*Diabetes distress* Intervention: 59.9 (22) Control: 42.3 (14) *HbA1c (%)* Intervention: 7.7 (1.0) Control: 7.6 (1.0)	*Diabetes distress*: Participants in the intervention group significant reported decrease in diabetes distress compared with the reported increase in the control group. *HbA1c (%)*: HbA1c levels decreased in both groups postintervention. The differences between the groups were not significant

^a^Duration of diabetes (IQR)

^b^Mean baseline scores ± standard error

^c^mean (95% CI)

CASM, Computer-Assisted Self-Management; CAPS, Computer-Assisted self-management with Problem Solving therapy; CES-D, Center for Epidemiological Studies-Depression scale; CBT, Cognitive Behavior Therapy; cRCT, Cluster Randomized Controlled Trial; HbA1c, Glycated Hemoglobin; LA, Leap Ahead; MI, Motivational Interviewing; PAID, Problem Areas in Diabetes; PST, Problem Solving Therapy; RCT, Randomized Controlled Trial; SD, Standard Deviation; T2DM, Type 2 Diabetes Mellitus

### Intervention characteristics

The intervention differed with regards to the type of non-specialists used, and in the nature of psychological intervention, duration and number of sessions. Among the included studies, psychological interventions were delivered by nurses [39, 40, 43, 50]; dietitians [37]; college graduates [38]; research assistants [35, 41]; undergraduate students [44]; peers i.e. diabetic patients [45] and community health workers [46, 47]. Welschen et al. [49] used dietitians and diabetes nurses to deliver the psychological intervention. Welch et al. [48] used diabetes educators. Interventionists in Dale et al. [36] were diabetes patients and diabetes nurses. Kim et al. [42] used nurses and community health workers to deliver their intervention. Most interventionists were health professionals (n = 7) or non-health professionals (n = 7).

Psychological treatments used in the interventions were cognitive behaviour therapy, motivational interviewing, problem solving therapy and mindfulness. Thirteen trials employed single psychological treatments in their interventions [35–41, 43–46, 48–50]. Three studies [37, 42, 47] incorporated two psychological treatments in their interventions. The frequency of intervention sessions ranged between 2 and 12 sessions, and duration of sessions ranged between 15 min and 2 h, over a period of 6 weeks to 24 months. Duration of intervention sessions was not reported for Dobler et al. [37] and Welschen et al. [49]. Gabbay et al. [39] did not report the frequency of sessions. Eight studies used individual intervention sessions [35–37, 39, 42, 44, 49, 50] and four studies used group sessions [41, 42, 46, 47]. Simmons et al. [45] used both individual and group intervention sessions. Three studies [38, 40, 48] did not specify the format of the intervention sessions. 14 studies reported the training of non-specialists by expert professionals. Two studies [42, 50] did not report the training of the non-specialist. In the control group, there were ten, four and two studies administering usual care, diabetes education and waiting list respectively.

### Outcomes

Five studies examined diabetes distress only, four studies examined depression only, seven studies measured both diabetes distress and depression and all 16 examined glycemic control. Diabetes distress was measured using DDS-17 in two studies [38, 45]; PAID-5 in one study [47] and PAID-20 in nine studies [35–37, 39, 40, 43, 46, 48, 50]. Depression was assessed using CES-D in five studies [35, 39, 41, 47, 49]; PHQ-8 in two studies [45, 47] and PHQ-9 in four studies [37, 42, 44, 46].

### Effect of non-specialist delivered psychological intervention on HbA1c

Eleven out of 16 RCTs included in the systematic review supplied sufficient data for meta-analysis. The meta-analysis produced an estimated effect size of − 0.13 for those randomised to a non-specialist delivered psychological intervention compared with the control group (95% CI − 0.22 to − 0.04, Z = 2.84, p = 0.005). There was high heterogeneity across the studies included in the meta-analysis (*I*^2^  = 71%, p = 0.0002). Excluding studies with multiple intervention arms (Dale et al. [36]; Welch et al. [48]) resulted in an increase in pooled HbA1c effect size from − 0.13 to − 0.24 (95% CI − 0.34 to − 0.14, p < 0.00001, *I* = 39%).

**Fig. 2:**
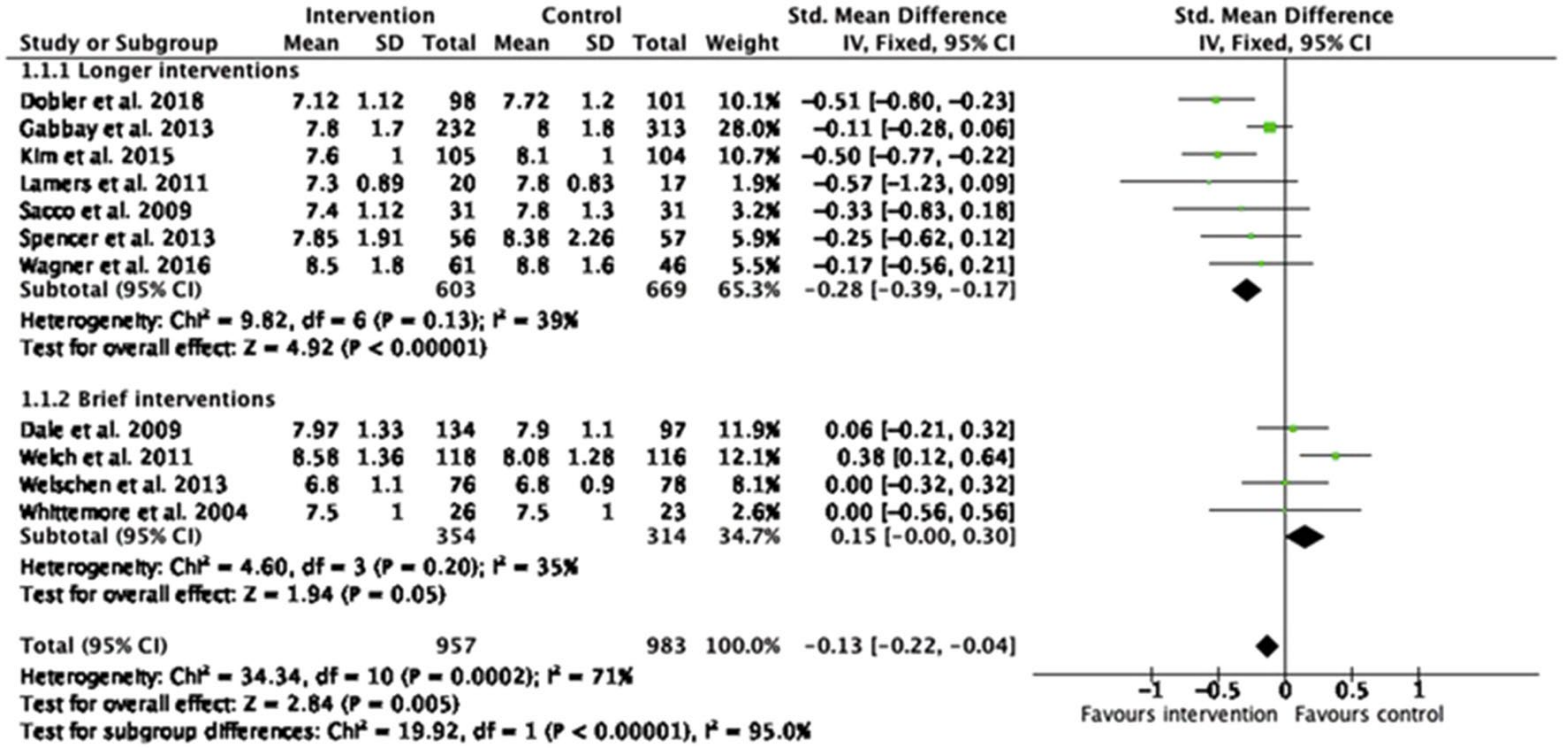
Forest plot for a random-effect meta-analysis of standardized mean difference in HbA1c comparing duration of non-specialist delivered psychological interventions

There were not enough studies to pool the effect sizes of different sub-categories of health professionals and non-health professionals. Comparison for different intervention providers indicated that non-health professionals delivered interventions seemed to have more favorable results than health professionals delivered interventions (SMD = − 0.24 95% CI − 0.47 to − 0.00, p = 0.05). Health professionals combined with non-health professionals showed an effect size of − 0.21 (95% CI − 0.40 to − 0.02, p = 0.03). Non-specialist delivered CBT interventions produced a non-significant effect of − 0.16 in HbA1c (95% CI − 0.41 to 0.09, p = 0.21). CBT combined with another psychological treatment had an effect size of − 0.39 (95% CI − 0.61 to − 0.16, p = 0.0007). Non-specialist delivered MI interventions had a non-significant effect size of − 0.01 (95% CI − 0.11 to 0.13, p = 0.87). MI combined with another psychological treatment had an effect size of − 0.51 (95% CI − 0.70 to − 0.31, p < 0.00001). Longer non-specialist delivered interventions (6 sessions and more) seemed to reduce HbA1c better (SMD = − 0.28, CI − 0.39 to − 0.17, p < 0.0001) in comparison with the brief interventions (SMD = 0.15, CI − 0.00 to 0.30, p = 0.05) (Fig. 2). In studies with participants with suboptimal glycemic control (HbA1c above 7%), there was no difference in overall effect size (SMD = − 0.14, CI − 0.24 to − 0.05, p = 0.003).

Of the studies that could not be pooled in the meta-analysis, one trial [35] reported significant decline in HbA1c levels at 3-month and at 8-month follow-up in participants with HbA1c levels less than 8% who had received CBT treatment.

### Effect of non-specialist delivered psychological intervention on diabetes distress

Given the varying measures used, results were not pooled in a meta-analysis. Of the twelve studies examining the impact on diabetes distress, four studies reported significant improvement in diabetes distress. Fisher et al. [38] reported that participants that received PST in addition to self-management education produced significantly greater reduction in diabetes distress relative to the other groups (p < 0.001; 392 participants). Even at 12-month follow-up this effect was sustained. In Spencer et al. [46] patients who received MI plus diabetes education significantly reduced diabetes distress compared to patients who received diabetes education alone (p < 0.05; 164 participants). Similarly, this was sustained at 6-month follow-up. In Whittemore et al. [50] 6 nurse-led sessions of MI plus self-management education resulted in significant decrease in diabetes distress (p < 0.01; 53 participants). The largest trial (n = 1299) by Simmons et al. [45] found that 8–12 peer-delivered individual sessions of MI tailored for T2DM patients were more effective than group sessions (− 0.42 95% CI − 0.75 to − 0.10). Conversely, in five interventions [36, 37, 39, 40, 48], there was no significant difference in diabetes distress score between the MI and control groups. Furthermore, studies [35, 43, 47] that used CBT either as single treatments or in conjunction with another psychological treatment did not significantly improve diabetes distress.

### Effect of non-specialist delivered psychological intervention on depression

The results for depressive symptom scores were not pooled in a meta-analysis as a result of the different scales used in measuring depression. Seven studies out of eleven reported significant improvements in depression. Gabbay et al. [39] reported that 2–9 MI sessions delivered by nurses significantly reduced depressive symptoms compared to those in the usual care group (p = 0.02). Inouye et al. [41] found that CBT delivered over 6 sessions by research assistants were effective in significantly improving symptoms of depression (p = 0.03). However, this effect was not sustained at 12 months follow-up (p-0.09). Similarly, in Sacco et al. [44] 9 undergraduate students-delivered sessions utilizing CBT techniques was found to significantly improve symptoms of depression (p < 0.005) whereas in Kim et al. [42], depression score in the waitlist control group decreased more than that in the intervention group. Welschen et al. [49] reported that patients who received 3–6 dietitians and diabetes nurses CBT sessions had significantly reduced symptoms of depression (p = 0.01). This effect was not sustained at follow-up (0.70). Wagner et al. [47] showed that 8 community health worker-led sessions of integrated care intervention involving techniques of dual therapy of CBT and mindfulness as well as diabetes education were effective in reducing depressive symptoms (p = 0.002). In Dobler et al. [37] MI combined with PST significantly improved depression symptoms. Conversely, in studies [35, 45, 46, 48] that used single psychological treatments of either CBT or MI, there was no significant difference in depression symptom score between the intervention and control groups.

### Risk of bias

Eight studies described a clear randomization process. Six studies did not provide enough information on the random sequence generation and were classified as unclear. Gabbay et al. [39] and Sacco et al. [44] were judged high risk of bias as they described a non-random component in the sequence generation process. Adequate concealment of allocations to intervention and control group was done in nine studies. Six studies reported insufficient information to permit judgement on this criterion. Sacco et al. [44] was judged high risk of bias as the method of allocation concealment could introduce bias.

Ten studies were assessed as high risk of performance and detection bias as the measurement of diabetes distress is likely to be influenced by lack of blinding. Chiu et al. [35] and Fisher et al. [38] were judged to report insufficient information on this criterion. Nine studies were assessed as high risk of performance and detection bias as the measurement of depression is likely to be influenced by lack of blinding. Chiu et al. [35] and Inouye et al. [41] were reported as having insufficient information on this criterion. Fifteen studies had low risk of performance and detection bias as measurement of HbA1c was not likely to be influenced by lack of blinding. Dobler et al. [37] yielded insufficient information to provide judgement on this criterion.

Eleven studies had low risk of reporting bias. Four studies were judged high risk of reporting bias. Heinrich et al. [40] had insufficient information to allow judgement on this criterion. Fourteen studies were judged to exhibit a low risk of attrition bias as the attrition in these studies did not affect outcomes, while Simmons et al. [45] and Wagner et al. [47] had attrition rates with possible impact on outcome data and were judged high risk.

## Discussion

We investigated the effects of non-specialist delivered psychological interventions on glycaemic control and mental health outcomes in individuals with T2DM. Although CBT and MI were commonly used, this review included trials of other psychological interventions delivered by non-specialists such as PST and mindfulness. A core mechanism in CBT and PST is the disclosure and subsequent reframing of negative thoughts and beliefs to achieve positive outcomes. MI facilitates expression of the individual’s beliefs, conflicts and barriers with the aim of stimulating behaviour change and adaptive coping. Overall, the review provides promising results with regards to the effect of non-specialist delivered psychological interventions on glycaemic control, depression and diabetes distress.

### Main findings

The 11 studies that were pooled in the meta-analysis demonstrated a reduction in mean HbA1c in favor of non-specialist delivered psychological interventions when compared with control groups and a significantly higher reduction was seen after trials with multiple intervention groups. Looking at the different types of non-specialist delivered psychological interventions, interventions that combined either CBT or MI with another psychological treatment showed the best improvement in HbA1c. Meta-analyses further showed that individuals living with T2DM might benefit more from non-specialist delivered psychological interventions with longer sessions with regard to the decrease of their HbA1c. The varying effect sizes highlighted in this review, from small to moderate, are likely to result in modest clinical improvements in glycemic control. These findings support the view of Chapman et al. [18] in using psychological interventions of CBT and MI for achieving clinically relevant benefits. The results from this review and Winkley et al. [23] are similar, in that, they report small effect in psychological interventions with participants with suboptimal glycemic control. There was a difference in the changes in glycaemic control when interventions were delivered by non-health professionals or health professionals combined with non-health professionals. This indicates that these types of interventions may hold possible benefits for persons living with T2DM in reducing the risk of onset and progression of T2DM related complications. However, the small number of studies warrant further research to know whether non-specialist delivered psychological interventions can sustain improvements in glycaemic control in individuals living with T2DM.

This review showed mixed results for diabetic distress in people with T2DM, with non-specialist delivered psychological interventions improving diabetes distress in some studies using college graduates, nurses, community health workers and diabetes peers, and reporting non-significant effect in others. With one exception [50], results from these four studies suggest that the improvements can be maintained over time, as Spencer et al. [44] and Fisher et al. [38] sustained the positive effects at 6-month and 12-month follow up respectively. The inconsistency of the effects of these interventions on diabetes distress could be the consequence of low intervention fidelity, insufficient intervention content addressing issues that are specific to living with diabetes and its management, personal perception of distress as well as mean diabetes distress score below cutoff point of the diabetes distress scales used. Although the results differ from the review by Schmidt et al. [51] who reported consistent effect of psychological interventions on diabetes distress, it should be noted that Schmidt et al. [51] reviewed studies that included individuals with type 1 and type 2 diabetes. Notably, the differential effect of non-specialist delivered psychological intervention was not observed in findings reported by Schmidt et al. [51] and thus, is a unique feature of this review.

There was more non-specialist delivered psychological interventions that had beneficial effects on depression than those achieved for diabetes distress. The improvement in depression symptoms were only observed in six studies that used students, research assistants, community health workers, dietitians and diabetes nurses. This differs from findings reported by Beres et al. [52] who found that six non-specialist delivered psychosocial interventions did not have any beneficial effect on depressive symptoms. It is noteworthy that the results of Beres et al. [52] are not consistent with the findings of this review as most of the interventions in Beres et al. [52] utilized psychoeducation rather than specific forms of psychological treatment such as CBT, MI or PST as was observed in this present review. The studies in this present review that reported improvements in depressive symptoms used CBT and this is congruent with other studies that reported the effects of CBT in treating depression in individuals with other chronic illnesses such as HIV/AIDS, stroke and chronic pain [53, 54]. Positive effects were sustained in Wagner et al. [47] at 3-month follow-up. However, these effects were not sustained at 6-month follow-up in Welschen et al. [49]; suggesting effectiveness of CBT in the short-term. It has been pointed out that depression is distinguished by persistent unhelpful thoughts that often results in feelings of guilt and low mood; hence the improved outcomes may be attributed to the role of CBT in identifying, disputing and changing unhelpful distortions (thoughts, feelings and behaviours).

In the studies under review, different non-specialists were used to deliver interventions. Unfortunately, important information related to non-specialists was often not reported including background, selection process and prior training. However, level of education was considered to be important in some studies, with attempts to recruit non-specialists with tertiary-level education such as research assistants, graduates and university students [35, 38, 41, 44]. Others sought to match non-specialists with the participants, for example on diabetes status; as peers or originating from the same community; community health workers [36, 45–47]. Likewise, other studies sought non-specialists that are directly involved in improving patient’s general health such as nurses and dietitians. [37, 39, 43, 48–50].

Furthermore, it was observed that the non-specialists with tertiary level education were trained in basic skills of CBT and PST, that are easy to learn and administer such as activity scheduling, behavioral experiments, reattribution and problem solving. Non-specialists directly involved in improving patient health underwent comprehensive training in MI and CBT including psychoeducation, with the shortest and longest duration of training being 2 days and 80 h respectively. Similarly, non-specialists selected on the basis of characteristics shared with participants also underwent comprehensive psychological treatment training (CBT, MI and mindfulness) including psychoeducation. Most studies explicitly stated that the training was conducted by specialist mental health professionals. Despite the variability in training duration, it has been suggested that training in psychological treatments has little influence on treatment effectiveness [55]. Wampold and Imel [56] acknowledged the importance of therapist characteristics such as empathy and adherence to intervention protocol in influencing treatment outcomes. The studies in this review did not explore any of these meaning that more attention needs to be paid to the attributes and qualities that make people adequate and appropriate non-specialist.

### Comparison with previous findings

The findings of the review suggest that psychological interventions delivered by non-specialists and aimed at older individuals with longer sessions may improve poor glycaemic control and mental health. More intense interventions (6 or more sessions) and those of longer duration (9 weeks or longer) were found to contribute to improved HbA1c levels and mental health outcomes. Brief interventions are likely to result in short-lived beneficial effects as observed in Welschen et al. [49] and Inuoye et al. [41]. Although this finding agrees with Sturt et al. [57] who reported that psychological interventions with intense sessions (6 or more) and longer duration (13 weeks or longer) decreases diabetes distress and HbA1c levels, this was recommended for delivery by specialists such as psychologists and psychiatrists. Neither the format of intervention sessions (individual or group) nor mode of delivery (face to face, web-based, phone-based) were found to influence the effectiveness of non-specialist delivered psychological interventions in spite of their relative advantages.

## Strength and limitations of this review

This review highlighted that all of the non-specialist delivered psychological interventions were conducted in high income countries even though they are needed in low- and middle-income countries, including sub-Saharan Africa, where specialist mental healthcare providers are scarce. The review identified an important age gap given that most of the participants in the included studies were over 50 years. This is concerning given that mortality resulting from co-occurrence of poor glycemic control and mental health problems also occurs in individuals below the age of 50. Despite this gap identified, there were some limitations in this review. The inclusion of randomized controlled trials, generally considered the gold standard of research in yielding the highest quality of evidence of the effectiveness of interventions, may have limited the scope of the evidence. It is possible that studies may have been missed given that randomized controlled trials are expensive to run and owing to limited resources, studies may have utilized inexpensive study designs such as observational studies and non-randomized controlled trials in illuminating knowledge related to non-specialist delivered psychological interventions.

The review highlighted variability of the interventions, outcomes as well as different types of non-specialists who vary with respect to their basic competencies and abilities to deliver intervention even with training. This prevented cross-comparison and quantitative analysis of the interventions’ effects on patient outcomes thus, precluding a comprehensive view of the effectiveness of non-specialist delivered psychological interventions. The review examined only English articles and exclusion of different languages may have influenced the results. Despite all the studies being RCTs, the overall evidence was of low-quality owing to the limitations in study design and implementation of the included trials, as well as the inconsistency of the effects across the included trials (see Appendix 3). Risk of bias assessments highlighted concerns about insufficient information on sequence generation and allocation concealment. However, given that included studies reported non-significant results, it is less likely that there is publication bias. Most of the studies did not satisfy the criterion that participants were blinded to treatment allocation and outcomes assessors, even though it is possible to blind outcome assessment. However, it should be noted that it is difficult to blind participants in psychological interventions [22]. The generalizability of the findings needs to be made with caution given that few trials that met the inclusion criteria, the majority of which had small sample sizes. Despite the low quality of evidence from the included studies, they are still informative of the potential effectiveness of non-specialist delivered psychological interventions, especially given the congruency of some of the results with effects of non-specialist delivered psychological interventions in the general population and other chronic conditions [31, 58].

## Conclusion

In individuals with T2DM, there is some beneficial effects of non-specialist delivered psychological intervention on glycemic control, depression and diabetes distress. However, this is based on a small number of studies with heterogeneous interventions and reporting of outcomes. Given the broad range of non-specialists, the literature does not yet support definitive recommendations about which specific non-specialist holds the most promise, highlighting the need for further research. The psychological interventions found in this review such as PST, CBT and MI have been recommended as psychological treatments for delivery by non-specialists in low- and middle-income countries, including sub-Saharan Africa [52, 59]. Despite the relevance of the findings of this review to these settings, more research needs to be done in low- and middle-income countries to provide more evidence of the potential effectiveness of non-specialist delivered psychological intervention for individuals living with T2DM as the studies included in this review all come from high income countries. In addition to qualitative research investigating the quality of the relationships between intervention providers and recipients, future interventions would benefit from comprehensive classification of non-specialists, including the psychological interventions they provide, in order to understand the basic competencies needed for successful delivery as well as to ensure availability of comparable and standardized interventions.

## Data Availability

Not applicable.

## Appendices

**Appendices contents:**

- Appendix 1: Systematic review search strategies
- Appendix 2: Data extraction form
- Appendix 3: Grade assessment
- Appendix 4: Forest plot for a random-effect meta-analysis of standardized mean difference in HbA1c comparing health professionals and non-health professionals

## Appendix 1: Systematic review search strategies

**EMBASE (Ovid SP)**

1. Paramedical Personnel/
2. Health Auxiliary/
3. Nursing Assistant/
4. Caregiver/
5. Voluntary Worker/
6. Self Help/
7. Social Support/
8. Health Care Manpower/
9. (lay adj3 (worker? or visitor? or attendant? or aide or aides or support* or person* or helper? or carer? or caregiver? or care giver? or consultant? or advisor? or counselor? or counsellor? or assistant? or staff)).ti,ab.
10. ((voluntary or volunteer?) adj3 (worker? or visitor? or attendant? or aide or aides or support* or person* or helper? or carer? or caregiver? or care giver? or consultant? or advisor? or counselor? or counsellor? or assistant? or staff)).ti,ab.
11. (untrained adj3 (worker? or visitor? or attendant? or aide or aides or support* or person* or helper? or carer? or caregiver? or care giver? or consultant? or advisor? or counselor? or counsellor? or assistant? or staff or nurse? or doctor? or physician? or therapist?)).ti,ab.
12. (trained adj3 (worker? or visitor? or attendant? or aide or aides or support* or person* or helper? or carer? or caregiver? or care giver? or consultant? or advisor? or counselor? or counsellor? or assistant? or staff or nurse? or doctor? or physician? or therapist?)).ti,ab.
13. (unlicensed adj3 (worker? or visitor? or attendant? or aide or aides or support* or person* or helper? or carer? or caregiver? or care giver? or consultant? or advisor? or counselor? or counsellor? or assistant? or staff or nurse? or doctor? or physician? or therapist?)).ti,ab.
14. ((nonprofessional? or non professional?) adj3 (worker? or visitor? or attendant? or aide or aides or support* or person* or helper? or carer? or caregiver? or care giver? or consultant? or advisor? or counselor? or counsellor? or assistant? or staff)).ti,ab.
15. ((non medical or non health or non healthcare or non health care) adj3 (worker? or visitor? or attendant? or aide or aides or support* or person* or helper? or carer? or caregiver? or care giver? or consultant? or advisor? or counselor? or counsellor? or assistant? or staff)).ti,ab.
16. (community adj3 (worker? or visitor? or attendant? or aide or aides or support* or person* or helper? or carer? or caregiver? or care giver? or consultant? or advisor? or counselor? or counsellor? or assistant? or staff)).ti,ab.
17. (paraprofessional? or paramedic or paramedics or paramedical worker? or paramedical personnel or allied health personnel or allied health worker? or support worker? or non specialist? or specially trained or barefoot doctor? or nurs* aid* or psychiatric aide? or psychiatric attendant? or social worker? or teacher? or school staff or trainer?).ti,ab.
18. ((health* or medical*) adj3 (auxiliary or auxiliaries)).ti,ab.
19. (nurs* adj1 (auxiliary or auxiliaries)).ti,ab.
20. (informal adj (caregiver? or care giver? or carer?)).ti,ab.
21. (self help group? or support group?).ti,ab.
22. ((social or psychosocial) adj (care or support)).ti,ab.
23. (village adj3 worker?).ti,ab.
24. community based.ti,ab.
25. (community adj3 intervention?).ti,ab.
26. community network?.ti,ab.
27. ((health or health care or healthcare) adj manpower).ti,ab.
28. human resources.ti,ab.
29. (task? adj3 shift*).ti,ab.
30. (staff* adj3 chang*).ti,ab.
31. 1 or 2 or 3 or 4 or 5 or 6 or 7 or 8 or 9 or 10 or 11 or 12 or 13 or 14 or 15 or 16 or 17 or 18 or 19 or 20 or 21 or 22 or 23 or 24 or 25 or 26 or 27 or 28 or 29 or 30
32. ((typ? 2 or typ? II or typ?2 or typ?II) adj3 diabet$).tw,ot.
33. (non insulin$ depend$ or noninsulin$ depend$ or non insulin? depend$).mp. or noninsulin?depend$.tw,ot.
34. diabetes mellitus/ or diabetes mellitus, type 2/
35. 32 or 33 or 34
36. (problem? area? adj3 diabetes).tw.
37. (diabet* adj12 distress*).tw.
38. (((diabet* adj3 specific) or related) adj3 stress).tw.
39. diabet* stress.tw.
40. psycho* stress$.tw.
41. emotion$ distr$.tw.
42. depress*.mp. [mp = title, abstract, heading word, drug trade name, original title, device manufacturer, drug manufacturer, device trade name, keyword, floating subheading word, candidate term word]
43. (((depressi$ adj3 disorder$) or depressi$) adj3 symptom$).tw,ot.
44. 36 or 37 or 38 or 39 or 40 or 41 or 42 or 43
45. 31 and 35 and 44
46. random*.tw. or clinical trial*.mp. or exp health care quality/
47. Randomized Controlled Trial/
48. Controlled Clinical Trial/
49. (randomised or randomized or randomly).ti,ab.
50. intervention*.ti,ab.
51. evaluat*.ti,ab.
52. control*.ti,ab.
53. effect?.ti,ab.
54. impact.ti,ab.
55. ((pretest or pre test) and (posttest or post test)).ti,ab.
56. 46 or 47 or 48 or 49 or 50 or 51 or 52 or 53 or 54 or 55
57. 45 and 56

**Cinahl (Ebscohost)**

**Table:**

S64	S34 AND S37 AND S54 AND S63
S63	S55 OR S56 OR S57 OR S58 OR S59 OR S60 OR S61 OR S62
S62	PT clinical trial
S61	MH “Pretest–Posttest Design+”
S60	MH “Clinical Trials”
S59	TI (randomis* or randomiz* or random* W0 allocat*) OR AB (randomis* or randomiz* or random* W0 allocat*)
S58	PT research
S57	TI (intervention* or controlled or control W0 group* or compare or compared or before N5 after or pre N5 post or pretest or “pre test” or posttest or “post test” or evaluat* or effect or impact or repeated W0 measur*) OR AB (intervention* or controlled or control W0 group* or compare or compared or before N5 after or pre N5 post or pretest or “pre test” or posttest or “post test” or evaluat* or effect or impact or repeated W0 measur*)
S56	MH “Randomized Controlled Trials”
S55	MH “prognosis+” OR MH “study design+” or random*
S54	S38 OR S39 OR S40 OR S41 OR S42 OR S43 OR S44 OR S45 OR S46 OR S47 OR S48 OR S49 OR S50 OR S51 OR S52 OR S53
S53	AB (depress* N3 disorder*)
S52	TI (depress* N3 disorder*)
S51	AB (“depress*”)
S50	TI (“depress*”)
S49	AB (“psych* stress”)
S48	TI (“psych* stress”)
S47	AB (“emotion* stress”)
S46	TI (“emotion* stress”)
S45	AB (“diabet* stress”)
S44	TI (“diabet* stress”)
S43	AB (diabet* N3 (specific OR related) N3 stress)
S42	TI (diabet* N3 (specific OR related) N3 stress)
S41	AB (diabet* N12 distress*)
S40	TI (diabet* N12 distress*)
S39	AB (“problem# area#” N3 diabetes)
S38	TI (“problem# area#” N3 diabetes)
S37	S35 OR S36
S36	TI type 2 diabetes or type 2 diabetes mellitus or t2dm
S35	AB (“type 2 diabetes*)
S34	S1 OR S2 OR S3 OR S4 OR S5 OR S6 OR S7 OR S8 OR S9 OR S10 OR S11 OR S12 OR S13 OR S14 OR S15 OR S16 OR S17 OR S18 OR S19 OR S20 OR S21 OR S22 OR S23 OR S24 OR S25 OR S26 OR S27 OR S28 OR S29 OR S30 OR S31 OR S32 OR S33
S33	TI staff* N3 chang* OR AB staff* N3 chang*
S32	TI ((task or tasks) N3 shift*) OR AB ((task or tasks) N3 shift*)
S31	TI “human resources” OR AB “human resources”
S30	TI ((health or healthcare) W0 manpower) OR AB ((health or healthcare) W0 manpower)
S29	TI community W0 network* OR AB community W0 network*
S28	TI community N3 intervention* OR AB community N3 intervention*
S27	TI “community based” OR AB “community based”
S26	TI village N3 worker* OR AB village N3 worker*
S25	TI ((social or psychosocial) W0 (care or support)) OR AB ((social or psychosocial) W0 (care or support))
S24	TI (“self help group” or “self help groups” or “support group” or “support groups”) OR AB (“self help group” or “self help groups” or “support group” or “support groups”)
S23	TI (informal W0 (caregiver* or “care giver” or “care givers” or carer*)) OR AB (informal W0 (caregiver* or “care giver” or “care givers” or carer*))
S22	TI (nurs* N1 (auxiliary or auxiliaries)) OR AB (nurs* N1 (auxiliary or auxiliaries))
S21	TI ((health* or medical*) N3 (auxiliary or auxiliaries)) OR AB ((health* or medical*) N3 (auxiliary or auxiliaries))
S20	TI (paraprofessional* or paramedic or paramedics or paramedical W0 worker* or paramedical W0 personnel or “allied health personnel” or “allied health worker” or “allied health workers” or support W0 worker* or non W0 specialist* or “specially trained” or barefoot W0 doctor* or nurs* W0 aide* or psychiatric W0 aide* or psychiatric W0 attendant* or social W0 worker* or teacher* or "school staff" or trainer*) OR AB (paraprofessional* or paramedic or paramedics or paramedical W0 worker* or parame…
S19	TI (community N3 (worker* or visitor* or attendant* or aide or aides or support* or person* or helper* or carer* or caregiver* or “care giver” or “care givers” or consultant* or advisor* or counselor* or counsellor* or assistant* or staff)) OR AB (community N3 (worker* or visitor* or attendant* or aide or aides or support* or person* or helper* or carer* or caregiver* or “care giver” or “care givers” or consultant* or advisor* or counselor* or counsellor* or assistant* or staff))
S18	TI ((“non medical” or “non health” or “non healthcare”) N3 (worker* or visitor* or attendant* or aide or aides or support* or person* or helper* or carer* or caregiver* or “care giver” or “care givers” or consultant* or advisor* or counselor* or counsellor* or assistant* or staff)) OR AB ((“non medical” or “non health” or “non healthcare”) N3 (worker* or visitor* or attendant* or aide or aides or support* or person* or helper* or carer* or caregiver* or “care giver” or “care givers” or consul…
S17	TI ((nonprofessional* or “non professional” or “non professionals”) N3 (worker* or visitor* or attendant* or aide or aides or support* or person* or helper* or carer* or caregiver* or "care giver" or “care givers” or consultant* or advisor* or counselor* or counsellor* or assistant* or staff)) OR AB ((nonprofessional* or “non professional” or “non professionals”) N3 (worker* or visitor* or attendant* or aide or aides or support* or person* or helper* or carer* or caregiver* or "care giver" or…
S16	TI (unlicensed N3 (worker* or visitor* or attendant* or aide or aides or support* or person* or helper* or carer* or caregiver* or “care giver” or “care givers” or consultant* or advisor* or counselor* or counsellor* or assistant* or staff or nurse* or doctor* or physician* or therapist*)) OR AB (unlicensed N3 (worker* or visitor* or attendant* or aide or aides or support* or person* or helper* or carer* or caregiver* or “care giver” or “care givers” or consultant* or advisor* or counselor* o…
S15	TI (trained N3 (worker* or visitor* or attendant* or aide or aides or support* or person* or helper* or carer* or caregiver* or “care giver” or “care givers” or consultant* or advisor* or counselor* or counsellor* or assistant* or staff or nurse* or doctor* or physician* or therapist*)) OR AB (trained N3 (worker* or visitor* or attendant* or aide or aides or support* or person* or helper* or carer* or caregiver* or “care giver” or “care givers” or consultant* or advisor* or counselor* or coun…
S14	TI (untrained N3 (worker* or visitor* or attendant* or aide or aides or support* or person* or helper* or carer* or caregiver* or “care giver” or “care givers” or consultant* or advisor* or counselor* or counsellor* or assistant* or staff or nurse* or doctor* or physician* or therapist*)) OR AB (untrained N3 (worker* or visitor* or attendant* or aide or aides or support* or person* or helper* or carer* or caregiver* or “care giver” or “care givers” or consultant* or advisor* or counselor* or …
S13	TI ((voluntary or volunteer*) N3 (worker* or visitor* or attendant* or aide or aides or support* or person* or helper* or carer* or caregiver* or “care giver” or “care givers” or consultant* or advisor* or counselor* or counsellor* or assistant* or staff)) OR AB ((voluntary or volunteer*) N3 (worker* or visitor* or attendant* or aide or aides or support* or person* or helper* or carer* or caregiver* or “care giver” or “care givers” or consultant* or advisor* or counselor* or counsellor* or as…
S12	TI (lay N3 (worker* or visitor* or attendant* or aide or aides or support* or person* or helper* or carer* or caregiver* or “care giver” or “care givers” or consultant* or advisor* or counselor* or counsellor* or assistant* or staff)) OR AB (lay N3 (worker* or visitor* or attendant* or aide or aides or support* or person* or helper* or carer* or caregiver* or “care giver” or “care givers” or consultant* or advisor* or counselor* or counsellor* or assistant* or staff))
S11	(MH “Home Health Aides”)
S10	(MH “Health Personnel, Unlicensed”)
S9	(MH “Personnel Staffing and Scheduling”)
S8	(MH “Health Manpower”)
S7	(MH “Support Groups”)
S6	(MH “Volunteer Workers”)
S5	(MH “Community Networks”)
S4	(MH “Caregivers”)
S3	(MH “Nursing Assistants”)
S2	(MH “Community Health Workers”)
S1	(MH “Allied Health Personnel”)

**Medline (Ovid SP)**

1. Allied Health Personnel/
2. Community Health Workers/
3. Nurses' Aides/
4. Psychiatric Aides/
5. Caregivers/
6. Voluntary Workers/
7. Community Networks/
8. exp Self Help Groups/
9. Social Support/
10. Personnel Staffing.mp. and Scheduling/ [mp = title, abstract, original title, name of substance word, subject heading word, floating sub-heading word, keyword heading word, organism supplementary concept word, protocol supplementary concept word, rare disease supplementary concept word, unique identifier, synonyms]
11. (lay adj3 (worker? or visitor? or attendant? or aide or aides or support* or person* or helper? or carer? or caregiver? or care giver? or consultant? or advisor? or counselor? or counsellor? or assistant? or staff)).ti,ab.
12. ((voluntary or volunteer?) adj3 (worker? or visitor? or attendant? or aide or aides or support* or person* or helper? or carer? or caregiver? or care giver? or consultant? or advisor? or counselor? or counsellor? or assistant? or staff)).ti,ab.
13. (untrained adj3 (worker? or visitor? or attendant? or aide or aides or support* or person* or helper? or carer? or caregiver? or care giver? or consultant? or advisor? or counselor? or counsellor? or assistant? or staff or nurse? or doctor? or physician? or therapist?)).ti,ab.
14. (trained adj3 (worker? or visitor? or attendant? or aide or aides or support* or person* or helper? or carer? or caregiver? or care giver? or consultant? or advisor? or counselor? or counsellor? or assistant? or staff or nurse? or doctor? or physician? or therapist?)).ti,ab.
15. (unlicensed adj3 (worker? or visitor? or attendant? or aide or aides or support* or person* or helper? or carer? or caregiver? or care giver? or consultant? or advisor? or counselor? or counsellor? or assistant? or staff or nurse? or doctor? or physician? or therapist?)).ti,ab.
16. ((nonprofessional? or non professional?) adj3 (worker? or visitor? or attendant? or aide or aides or support* or person* or helper? or carer? or caregiver? or care giver? or consultant? or advisor? or counselor? or counsellor? or assistant? or staff)).ti,ab.
17. ((non medical or non health or non healthcare or non health care) adj3 (worker? or visitor? or attendant? or aide or aides or support* or person* or helper? or carer? or caregiver? or care giver? or consultant? or advisor? or counselor? or counsellor? or assistant? or staff)).ti,ab.
18. (community adj3 (worker? or visitor? or attendant? or aide or aides or support* or person* or helper? or carer? or caregiver? or care giver? or consultant? or advisor? or counselor? or counsellor? or assistant? or staff)).ti,ab.
19. (paraprofessional? or paramedic or paramedics or paramedical worker? or paramedical personnel or allied health personnel or allied health worker? or support worker? or non specialist? or specially trained or barefoot doctor? or nurs* aid* or psychiatric aide? or psychiatric attendant? or social worker? or teacher? or school staff or trainer?).ti,ab.
20. ((health* or medical*) adj3 (auxiliary or auxiliaries)).ti,ab.
21. (nurs* adj1 (auxiliary or auxiliaries)).ti,ab.
22. (informal adj (caregiver? or care giver? or carer?)).ti,ab.
23. (self help group? or support group?).ti,ab.
24. ((social or psychosocial) adj (care or support)).ti,ab.
25. (village adj3 worker?).ti,ab.
26. community based.ti,ab.
27. (community adj3 intervention?).ti,ab.
28. community network?.ti,ab.
29. ((health or health care or healthcare) adj manpower).ti,ab.
30. human resources.ti,ab.
31. (task? adj3 shift*).ti,ab.
32. (staff* adj3 chang*).ti,ab.
33. Health Manpower/
34. 1 or 2 or 3 or 4 or 5 or 6 or 7 or 8 or 9 or 10 or 11 or 12 or 13 or 14 or 15 or 16 or 17 or 18 or 19 or 20 or 21 or 22 or 23 or 24 or 25 or 26 or 27 or 28 or 29 or 30 or 31 or 32 or 33
35. diabetes mellitus/ or diabetes mellitus, type 2/
36. ((typ? 2 or typ? II or typ?2 or typ?II) adj3 diabet$).tw,ot.
37. (non insulin$ depend$ or noninsulin$ depend$ or non insulin? depend$).mp. or noninsulin?depend$.tw,ot.
38. 35 or 36 or 37
39. (problem? area? adj3 diabetes).tw.
40. (diabet* adj12 distress*).tw.
41. (((diabet* adj3 specific) or related) adj3 stress).tw.
42. diabet* stress.tw.
43. psycho* stress$.tw.
44. emotion$ distr$.tw.
45. depress*.mp. [mp = title, abstract, original title, name of substance word, subject heading word, floating sub-heading word, keyword heading word, organism supplementary concept word, protocol supplementary concept word, rare disease supplementary concept word, unique identifier, synonyms]
46. (((depressi$ adj3 disorder$) or depressi$) adj3 symptom$).tw,ot.
47. 39 or 40 or 41 or 42 or 43 or 44 or 45 or 46
48. Random* Control* Trial*.mp. [mp = title, abstract, original title, name of substance word, subject heading word, floating sub-heading word, keyword heading word, organism supplementary concept word, protocol supplementary concept word, rare disease supplementary concept word, unique identifier, synonyms]
49. Random* Control* Trial*.ti.
50. (Clinical Trials or Controlled Clinical Trial*).mp. [mp = title, abstract, original title, name of substance word, subject heading word, floating sub-heading word, keyword heading word, organism supplementary concept word, protocol supplementary concept word, rare disease supplementary concept word, unique identifier, synonyms]
51. ((Random* adj2 sampl*) or allocat*).mp. [mp = title, abstract, original title, name of substance word, subject heading word, floating sub-heading word, keyword heading word, organism supplementary concept word, protocol supplementary concept word, rare disease supplementary concept word, unique identifier, synonyms]
52. ((Random* adj Clinical Trial*) or Controlled Clinical Trial*).mp. [mp = title, abstract, original title, name of substance word, subject heading word, floating sub-heading word, keyword heading word, organism supplementary concept word, protocol supplementary concept word, rare disease supplementary concept word, unique identifier, synonyms]
53. evaluat*.ti,ab.
54. effect?.ti,ab.
55. impact.ti,ab.
56. trial.ti,ab.
57. ((pretest or pre test) and (posttest or post test)).ti,ab.
58. ((multicenter or multicentre or multi center or multi centre) adj study).ti,ab.
59. repeated measure*.ti,ab.
60. 34 and 38 and 47
61. 48 or 49 or 50 or 51 or 52 or 53 or 54 or 55 or 56 or 57 or 58 or 59
62. 60 and 61

**PsycINFO (Ovid SP)**

1. Allied Health Personnel/
2. Nonprofessional Personnel/
3. Paraprofessional Personnel/
4. Psychiatric Aides/
5. Home Care Personnel/
6. Caregivers/
7. Volunteers/
8. Support Groups/
9. Social Support/
10. (lay adj3 (worker? or visitor? or attendant? or aide or aides or support* or person* or helper? or carer? or caregiver? or care giver? or consultant? or advisor? or counselor? or counsellor? or assistant? or staff)).ti,ab.
11. ((voluntary or volunteer?) adj3 (worker? or visitor? or attendant? or aide or aides or support* or person* or helper? or carer? or caregiver? or care giver? or consultant? or advisor? or counselor? or counsellor? or assistant? or staff)).ti,ab.
12. (untrained adj3 (worker? or visitor? or attendant? or aide or aides or support* or person* or helper? or carer? or caregiver? or care giver? or consultant? or advisor? or counselor? or counsellor? or assistant? or staff or nurse? or doctor? or physician? or therapist?)).ti,ab.
13. (trained adj3 (worker? or visitor? or attendant? or aide or aides or support* or person* or helper? or carer? or caregiver? or care giver? or consultant? or advisor? or counselor? or counsellor? or assistant? or staff or nurse? or doctor? or physician? or therapist?)).ti,ab.
14. (unlicensed adj3 (worker? or visitor? or attendant? or aide or aides or support* or person* or helper? or carer? or caregiver? or care giver? or consultant? or advisor? or counselor? or counsellor? or assistant? or staff or nurse? or doctor? or physician? or therapist?)).ti,ab.
15. ((nonprofessional? or non professional?) adj3 (worker? or visitor? or attendant? or aide or aides or support* or person* or helper? or carer? or caregiver? or care giver? or consultant? or advisor? or counselor? or counsellor? or assistant? or staff)).ti,ab.
16. ((non medical or non health or non healthcare or non health care) adj3 (worker? or visitor? or attendant? or aide or aides or support* or person* or helper? or carer? or caregiver? or care giver? or consultant? or advisor? or counselor? or counsellor? or assistant? or staff)).ti,ab.
17. (community adj3 (worker? or visitor? or attendant? or aide or aides or support* or person* or helper? or carer? or caregiver? or care giver? or consultant? or advisor? or counselor? or counsellor? or assistant? or staff)).ti,ab.
18. (paraprofessional? or paramedic or paramedics or paramedical worker? or paramedical personnel or allied health personnel or allied health worker? or support worker? or non specialist? or specially trained or barefoot doctor? or nurs* aid* or psychiatric aide? or psychiatric attendant? or social worker? or teacher? or school staff or trainer?).ti,ab.
19. ((health* or medical*) adj3 (auxiliary or auxiliaries)).ti,ab.
20. (nurs* adj1 (auxiliary or auxiliaries)).ti,ab.
21. (informal adj (caregiver? or care giver? or carer?)).ti,ab.
22. (self help group? or support group?).ti,ab.
23. ((social or psychosocial) adj (care or support)).ti,ab.
24. (village adj3 worker?).ti,ab.
25. community based.ti,ab.
26. (community adj3 intervention?).ti,ab.
27. community network?.ti,ab.
28. ((health or health care or healthcare) adj manpower).ti,ab.
29. human resources.ti,ab.
30. (task? adj3 shift*).ti,ab.
31. (staff* adj3 chang*).ti,ab.
32. 1 or 2 or 3 or 4 or 5 or 6 or 7 or 8 or 9 or 10 or 11 or 12 or 13 or 14 or 15 or 16 or 17 or 18 or 19 or 20 or 21 or 22 or 23 or 24 or 25 or 26 or 27 or 28 or 29 or 30 or 31
33. diabetes mellitus/ or diabetes mellitus, type 2/
34. ((typ? 2 or typ? II or typ?2 or typ?II) adj3 diabet$).tw,ot.
35. (non insulin$ depend$ or noninsulin$ depend$ or non insulin? depend$).mp. or noninsulin?depend$.tw,ot.
36. 33 or 34 or 35
37. (problem? area? adj3 diabetes).tw.
38. (diabet* adj12 distress*).tw.
39. (((diabet* adj3 specific) or related) adj3 stress).tw.
40. diabet* stress.tw.
41. psycho* stress$.tw.
42. emotion$ distr$.tw.
43. depress*.mp. [mp = title, abstract, heading word, table of contents, key concepts, original title, tests & measures]
44. (((depressi$ adj3 disorder$) or depressi$) adj3 symptom$).tw,ot.
45. 37 or 38 or 39 or 40 or 41 or 42 or 43 or 44
46. (control* or random*).tw. or exp Treatment/
47. ((pretest or pre test) and (posttest or post test)).ti,ab.
48. ((multicenter or multicentre or multi center or multi centre) adj study).ti,ab.
49. repeated measure*.ti,ab.
50. (randomised or randomized or randomly allocated or random allocation or control* or evaluat* or effect? or impact or intervention*).ti,ab.
51. 46 or 47 or 48 or 49 or 50
52. 32 and 36 and 45
53. 51 and 52

**Cochrane central**

1. [mh “Allied Health Personnel”]
2. [mh “Community Health Workers”]
3. [mh “Nurses' Aides”]
4. [mh “Psychiatric Aides”]
5. [mh “Caregivers”]
6. [mh “Voluntary Workers”]
7. [mh “Community Networks”]
8. [mh “Self-Help Groups”]
9. [mh “Health Manpower”]
10. [mh “Personnel Staffing and Scheduling”]
11. [mh “Social Support”]
12. (lay NEAR/3 (worker* or visitor* or attendant* or aide or aides or support* or person* or helper* or carer* or caregiver* or “care giver” or “care givers” or consultant* or advisor* or counselor* or counsellor* or assistant* or staff)):ti,ab
13. ((voluntary or volunteer*) NEAR/3 (worker* or visitor* or attendant* or aide or aides or support* or person* or helper* or carer* or caregiver* or “care giver” or “care givers” or consultant* or advisor* or counselor* or counsellor* or assistant* or staff)):ti,ab
14. (untrained NEAR/3 (worker* or visitor* or attendant* or aide or aides or support* or person* or helper* or carer* or caregiver* or “care giver” or “care givers” or consultant* or advisor* or counselor* or counsellor* or assistant* or staff or nurse* or doctor* or physician* or therapist*)):ti,ab
15. (trained NEAR/3 (worker* or visitor* or attendant* or aide or aides or support* or person* or helper* or carer* or caregiver* or “care giver” or “care givers” or consultant* or advisor* or counselor* or counsellor* or assistant* or staff or nurse* or doctor* or physician* or therapist*)):ti,ab
16. (unlicensed NEAR/3 (worker* or visitor* or attendant* or aide or aides or support* or person* or helper* or carer* or caregiver* or “care giver” or “care givers” or consultant* or advisor* or counselor* or counsellor* or assistant* or staff or nurse* or doctor* or physician* or therapist*)):ti,ab
17. ((nonprofessional* or “non professional” or “non professionals”) NEAR/3 (worker* or visitor* or attendant* or aide or aides or support* or person* or helper* or carer* or caregiver* or “care giver” or “care givers” or consultant* or advisor* or counselor* or counsellor* or assistant* or staff)):ti,ab
18. ((“non medical” or “non health” or “non healthcare” or “non health care”) NEAR/3 (worker* or visitor* or attendant* or aide or aides or support* or person* or helper* or carer* or caregiver* or “care giver” or “care givers” or consultant* or advisor* or counselor* or counsellor* or assistant* or staff)):ti,ab
19. (community NEAR/3 (worker* or visitor* or attendant* or aide or aides or support* or person* or helper* or carer* or caregiver* or “care giver” or “care givers” or consultant* or advisor* or counselor* or counsellor* or assistant* or staff)):ti,ab
20. (paraprofessional* or paramedic or paramedics or “paramedical worker” or “paramedical workers” or “paramedical personnel” or “allied health personnel” or “allied health worker” or “allied health workers” or support NEXT worker* or non NEXT specialist* or “specially trained” or barefoot NEXT doctor* or nurse* NEXT aide* or psychiatric NEXT aide* or psychiatric NEXT attendant* or social NEXT worker* or teacher* or “school staff” or trainer*):ti,ab
21. ((health* or medical*) NEAR/3 (auxiliary or auxiliaries)):ti,ab
22. (nurse* NEAR/1 (auxiliary or auxiliaries)):ti,ab
23. (informal NEXT (caregiver* or “care giver” or “care givers” or carer*)):ti,ab
24. (“self help group” or “self help groups” or “support group” or "support groups"):ti,ab
25. ((social or psychosocial) NEXT (care or support)):ti,ab
26. (village NEAR/3 worker*):ti,ab
27. “community based”:ti,ab
28. (community NEAR/3 intervention*):ti,ab
29. (“community network” or “community networks”):ti,ab
30. ((health or “health care” or healthcare) NEXT manpower):ti,ab
31. “human resources”:ti,ab
32. (task NEAR/3 shift* or taskshift*):ti,ab
33. (staff* NEAR/3 chang*):ti,ab
34. (#1 OR #2 OR #3 OR #4 OR #5 OR #6 OR #7 OR #8 OR #9 OR #10 OR #11 OR #12 OR #13 OR #14 OR #15 OR #16 OR #17 OR #18 OR #19 OR #20 OR #21 OR #22 OR #23 OR #24 OR #25 OR #26 OR #27 OR #28 OR #29 OR #30 OR #31 OR #32 OR #33)
35. (type 2 diabetes*):ti,ab,kw
36. (“non insulin” NEXT (depend*)):ti,ab,kw
37. (type II diabetes*):ti,ab,kw
38. (#35 OR #36 OR #37)
39. ((problem* next area*) near/4 “diabetes”):ti,ab,kw
40. (diabet* near/13 distress*):ti,ab,kw
41. (diabet* near/4 (“specific” or “related”) near/4 “stress”):ti,ab,kw
42. (diabet* next “stress”):ti,ab,kw
43. (psych* next “stress”):ti,ab,kw
44. (emotion* next “distress”):ti,ab,kw
45. (depress*):ti,ab,kw
46. (depress* next disorder):ti,ab,kw
47. (#39 OR #40 OR #41 OR #42 OR #43 OR #44 OR #45 OR #46)
48. #34 AND #38 AND #47

**WHO Global Health Library**

((non and specialist* and health* and worker*) or (nonprofessional* and health* and worker*) or (non and professional* and health* and worker*) or (untrained and health* and worker*) or (unlicensed and health* and worker*) or (lay and health* and worker*) or (voluntary and health* and worker*) or (volunteer* and health* and worker*) or (community and health* and worker*) or (paraprofessional* and health* and worker*) or (informal and health* and worker*) or (village and health* and worker*) or (non and specialist* and health* and personnel) or (nonprofessional* and health* and personnel) or (non and professional* and health* and personnel) or (untrained and health* and personnel) or (unlicensed and health* and personnel) or (lay and health* and personnel) or (voluntary and health* and personnel) or (volunteer* and health* and personnel) or (community and health* and personnel) or (paraprofessional* and health* and personnel) or (informal and health* and personnel) or (village and health* and personnel) or (non and specialist* and health* and carer*) or (nonprofessional* and health* and carer*) or (non and professional* and health* and carer*) or (untrained and health* and carer*) or (unlicensed and health* and carer*) or (lay and health* and carer*) or (voluntary and health* and carer*) or (volunteer* and health* and carer*) or (community and health* and carer*) or (paraprofessional* and health* and carer*) or (informal and health* and carer*) or (village and health* and carer*) or (non and specialist* and health* and caregiver*) or (nonprofessional* and health* and caregiver*) or (non and professional* and health* and caregiver*) or (untrained and health* and caregiver*) or (unlicensed and health* and caregiver*) or (lay and health* and caregiver*) or (voluntary and health* and caregiver*) or (volunteer* and health* and caregiver*) or (community and health* and caregiver*) or (paraprofessional* and health* and caregiver*) or (informal and health* and caregiver*) or (village and health* and caregiver*) or (non and specialist* and health* and (care and giver*)) or (nonprofessional* and health* and (care and giver*)) or (non and professional* and health* and (care and giver*)) or (untrained and health* and (care and giver*)) or (unlicensed and health* and (care and giver*)) or (lay and health* and (care and giver*)) or (voluntary and health* and (care and giver*)) or (volunteer* and health* and (care and giver*)) or (community and health* and (care and giver*)) or (paraprofessional* and health* and (care and giver*)) or (informal and health* and (care and giver*)) or (village and health* and (care and giver*)) or (non and specialist* and health* and provider*) or (nonprofessional* and health* and provider*) or (non and professional* and health* and provider*) or (untrained and health* and provider*) or (unlicensed and health* and provider*) or (lay and health* and provider*) or (voluntary and health* and provider*) or (volunteer* and health* and provider*) or (community and health* and provider*) or (paraprofessional* and health* and provider*) or (informal and health* and provider*) or (village and health* and provider*) or (social and worker*) or teacher* or (school and staff) or (self and help and group*) or (support and group*) or (task* and shift*) or taskshift* or (health* and manpower) or (human and resources)) AND ((typ* 2 diabet*) or (typ* II diabet*) or (non insulin dependent*))) AND (tw:((problem area diabet*) or (diabet* distress*) or (diabet* stress) or (psych* *stress) or (emotion* distress*))) AND ((depress*) or (depress* disorder*)) AND (randomiz* or randomis* or (controlled and trial) or (multicenter and study) or (multicentre and study) or (cluster and trial) or (controlled and before and after) or pretest or (pre and test) or posttest or (post and test) or intervention* or evaluat* or effect or impact or (time and series) or (time and points) or (repeated and measure*).

**WHO (ICTRP)**

diabet* AND distress OR diabet* AND problem areas OR depress* AND depress* disorder.

**ClinicalTrials.gov**

(diabetes OR diabetic) AND (distress OR problem areas) AND (depression OR depressive disorder).

Age: 18–65+.

## Appendix 2: Data extraction form

Study design, Country:

Mean age of participants in

years (SD), % of males and females:

Sample size/N of intervention group

/N of control group:

Duration of T2DM [mean years

(SD)]:

Cadre of non-specialist (mode of

delivery):

Intervention description

(follow-up):

Outcomes measures of relevance

[Mean baseline scores (SD)]

Results:

## Appendix 3: Checklist to aid consistency and reproducibility of GRADE assessments

**Table:**

	Glycemic control	Depression	Diabetes distress
Risk of bias (Serious limitations)	Allocation concealment unclear in 3 studies. Allocation concealment bias high in 1 study Objective outcome was used. Blinding bias for HbA1c unclear in 1 study Selective reporting bias high in 4 studies Attrition bias high in 2 studies	Allocation concealment unclear in 2 studies No blinding for subjective outcomes; participants and investigators not blinded Attrition bias high in 2 studies Selective reporting bias high in 2 studies	Allocation concealment unclear in 2 studies No blinding for subjective outcomes; participants and investigators not blinded Attrition bias high in 2 studies Selective reporting bias high in 4 studies
Indirectness (Not serious)	The patients/population and comparators in the studies all provide direct evidence to the review question at hand. The severity of outcomes (depression and diabetes distress) was assessed using different scales in different trials. HbA1c was a surrogate outcome but was not marked down as it is closely related to changes in patient important outcomes for diabetic individuals. Evidence was judged to have no serious indirectness but variability in intervention and outcome measure was noted		
Imprecision (Not serious)	With the total number of patients included in all the trials 3564 and trials reporting small and moderate reductions and other trials reporting non-significant results, evidence was judged to be borderline imprecise however, not enough to downgrade the results as only two studies enrolled a small number of participants		
Inconsistency (Serious)	Evidence was judged to have serious inconsistency as the direction and magnitude of effect varied across the different trials, with mixed results in diabetes distress		
Publication bias (Unlikely)	Negative and positive studies were published and search for studies was comprehensive		

The body of high-quality evidence of RCTs necessitate downgrading to low quality because of risk of bias and inconsistency.

## Abbreviations

AIDS: Acquired Immune Deficiency Syndrome
CASM: Computer-Assisted Self-Management
CAPS: Computer-Assisted self-management with Problem Solving therapy
CBT: Cognitive Behavior Therapy
CCT: Client Centered Therapy
CES-D: Center for Epidemiological Studies-Depression scale
CI: Confidence Interval
cRCT: Cluster Randomized Controlled Trial
DDS: Diabetes Distress Scale
GRADE: Grading of Recommendations, Assessment, Development and Evaluations
LA: Leap Ahead
HbA1c: Glycated Hemoglobin
HIV: Human Immunodeficiency Virus
MI: Motivational Interviewing
PAID: Problem Areas in Diabetes
PRISMA: Preferred Reporting Items for Systematic reviews and Meta-Analyses
PHQ: Patient Health Questionnaire
PST: Problem Solving Therapy
RCT: Randomized Controlled Trials
SD: Standard Deviation
SMD: Standardized Mean Difference
T2DM: Type 2 Diabetes Mellitus
UK: United Kingdom
USA: United States of America
